# Positive Intraoperative Cultures in Cup Revisions of THA: What Happens to the Stem?

**DOI:** 10.1097/CORR.0000000000002873

**Published:** 2023-10-12

**Authors:** Karsten D. Ottink, Desirée M. J. Dorleijn, Willemijn Spierenburg, Joris J. W. Ploegmakers, Wierd P. Zijlstra, Harmen E. Ettema, Bas L. E. F. ten Have, Paul C. Jutte, Marjan Wouthuyzen-Bakker

**Affiliations:** 1 Department of Orthopedics, University Medical Center Groningen, University of Groningen, Groningen, the Netherlands; 2 Department of Orthopedics, Medical Center Leeuwarden, Leeuwarden, the Netherlands; 3 Department of Orthopedics, Isala, Zwolle, the Netherlands; 4 Department of Orthopedics, Martini Hospital, Groningen, the Netherlands; 5 Department of Medical Microbiology and Infection Prevention, University Medical Center Groningen, University of Groningen, Groningen, the Netherlands

## Abstract

**Background:**

Positive intraoperative cultures (PICs) are encountered in some patients undergoing revision of the acetabular cup after a previous THA. It is unknown whether PIC of the cup indicates whether the stem is infected as well and what happens to the stem during follow-up.

**Questions/purposes:**

(1) What proportion of patients undergoing THA who undergo cup revision have PICs? (2) What is the survival of the stem during follow-up in cup revisions with PICs versus that of those with negative cultures? (3) Does antibiotic treatment of PIC of the cup prevent revision THA during follow-up?

**Methods:**

In this retrospective, comparative multicenter study, five surgeons at four centers performed 338 acetabular cup revisions between January 2015 and December 2017. After evaluating the data, we excluded one patient because of an incomplete dataset and 77 patients because fewer than three intraoperative cultures were obtained during surgery, leaving 260 patients for analysis. Follow-up was 2 years. Patients were stratified into three cohorts: no PIC, one PIC, and two or more PICs.

**Results:**

The proportion of patients with one or more PIC was 15% (39 of 260). A total of 8% (21 of 260) had one and 7% (18 of 260) had two or more PICs. Stem survival was lower in patients with two or more PICs, but stem revision for periprosthetic joint infection was similar between groups. Two-year survival, which was defined as freedom from revision for any cause or infection, was 97% (95% confidence interval 95% to 99%) in the group without PICs, 100% (95% CI 95% to 100%) in the group with one PIC, and 86% (95% CI 68% to 100%; p = 0.08) in the group with two or more PICs. None of the patients in the no PIC and one PIC groups were treated with antibiotics. In the two or more PICs cohort, 12 of 18 patients were treated. The stem survived in one of 12 patients treated with antibiotics versus two of six patients who were not treated with antibiotics.

**Conclusion:**

When treated with antibiotics, more than two PICs isolated during cup revision surgery do not have a major impact on survival of the stem during follow-up. A larger cohort of patients with PICs during cup revision might confirm these findings.

**Level of Evidence:**

Level III, therapeutic study.

## Introduction

Up to 4.6% of patients with THA undergo revision within 12 years after primary THA in the Netherlands [5]. Loosening of the prosthesis is the most common cause of revision surgery [4]. In approximately 10% to 15% of presumed aseptic hip revisions, intraoperative cultures are positive [3, 8, 11-13]. In these cases, patients often had inadequate surgical debridement; additionally, these unexpectedly positive cultures often resulted in a delay of antibiotic treatment. Although one might expect a higher failure rate of the implant during follow-up, studies are inconsistent [6, 8]. Partial revision is a risk factor for failure in patients with unexpectedly positive cultures during revision surgery [6]. A partial revision of the acetabular component of the hip (cup revision) may be indicated for multiple reasons, most often septic or aseptic loosening, malposition, or dislocation and fracture. In case of positive cultures during cup revision, it is often unclear whether the stem is infected as well and whether patients should be treated with lifelong suppressive antibiotic treatment to prevent stem failure during follow-up.

We therefore asked: (1) What proportion of patients undergoing THA who undergo cup revision have positive intraoperative cultures (PICs)? (2) What is the survival of the stem during follow-up in cup revisions with PIC versus that of those with negative cultures? (3) Does antibiotic treatment of PIC of the cup prevent revision THA during follow-up?

## Patients and Methods

### Study Design and Setting

This study was a retrospective, comparative, multicenter study. Four hospitals participating in the Northern Infection Network Joint Arthroplasty in the Netherlands participated in this study. The participating hospitals were the University Medical Center Groningen, a university hospital, and three large peripheral referral hospitals: Martini Hospital, Medical Center Leeuwarden, and the Isala Clinics. In each center, approximately eight orthopaedic surgeons are active, with two surgeons at each center dedicated to hip revision surgery.

### Participants

We included patients ≥ 18 years old who underwent isolated cup revision without revision of the stem from January 1, 2015, to December 31, 2017. The follow-up period after cup revision was at least 2 years. Exclusion criteria were patients in whom fewer than three intraoperative cultures were obtained during cup revision, because in these patients, an infection with negative cultures cannot be ruled in [1]; we also excluded patients with a follow-up period of less than 2 years and those with incomplete data.

All procedures were performed by a hip reconstruction surgeon (JJWP, PCJ, WPZ, HEE, BLEFtH) in a Class 1 laminar airflow–fitted operating room, and standard perioperative antimicrobial prophylaxis (2 grams of intravenous cefazolin) was administered.

During cup revision, deep tissue cultures were obtained at the surgeon’s discretion from different anatomic locations, and for each biopsy, new sterile instruments were used. Each sample was cultured for 9 to 11 days on blood and chocolate agar under aerobic conditions (with 5% CO_2_) and on Brucella blood agar under anaerobic conditions. In addition, all samples were cultured in fastidious broth. The cup was sonicated if sonication was available in the participating center. Empirical antibiotic treatment was started after cultures were obtained in cases of suspected infection.

### Descriptive Data

**Fig. 1:**
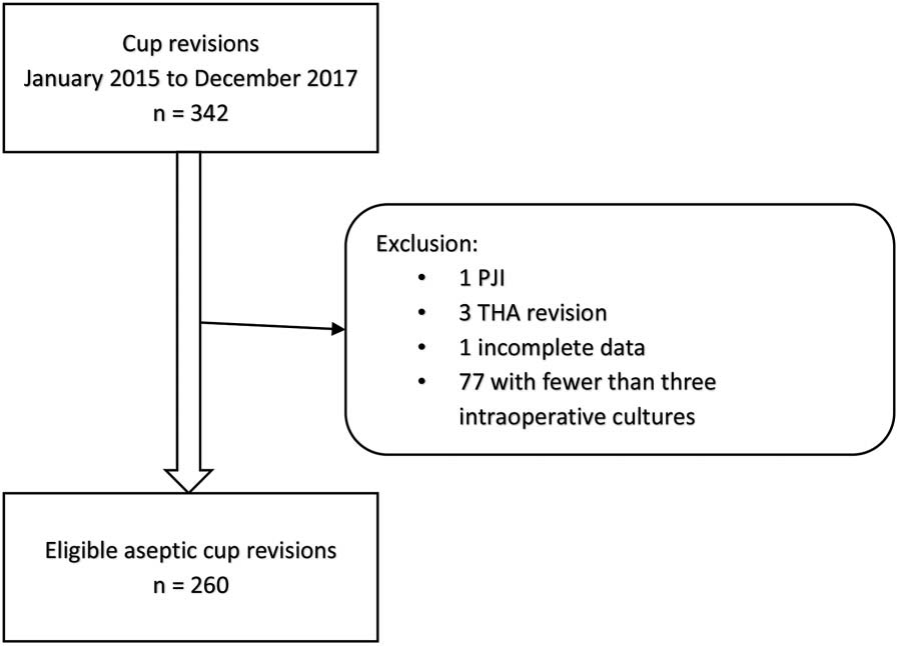
This flowchart represents eligible patients with aseptic cup revision in four hospitals associated with the Northern Infection Network Joint Arthroplasty. PJI = periprosthetic joint infection.

A total of 338 cup revisions were evaluated. One patient was excluded because an insufficient amount of data were collected, and 77 patients were excluded because an insufficient number of intraoperative cultures were obtained during cup revision. A total of 260 patients were included in the final analysis (Fig. 1). The median age of patients during cup revision was 74 years (range 30 to 97 years), and 68% (177 of 260) were female (Table 1).

**Table 1.** Demographic details of patients who underwent cup revision for aseptic reasons at four hospitals in the NINJA network between 2015 and 2017

Variable	No UPIC (85% [n = 221])	One UPIC (8% [n = 21])	Two or more UPICs (7% [n = 18])	p value
Age in years at cup revision, median (IQR)	74 (12)	76 (13)	75 (14)	0.89^a^
Female, % (n)	68% (151)	67% (14)	67% (12)	0.98^b^
C-reactive protein in mg/L, median (IQR)	2.4 (4)	3.5 (8.8)	18.8 (36.7)	0.09^a^
Erythrocyte sedimentation rate in mm/hour, median (IQR)	13 (16)	12 (16)	17 (50)	0.57^a^
Comorbidities, % (n)				
Smoking	7% (16)	5% (1)	0% (0)	0.46^b^
Diabetes	13% (29)	10% (2)	28% (5)	0.20^b^
Renal failure (eGFR < 30 ml/min)	3% (7)	5% (1)	0% (0)	0.68^b^
Gout	2% (4)	10% (2)	6% (1)	0.09^b^
Rheumatoid arthritis	4% (8)	5% (1)	0% (0)	0.68^b^
Using immunosuppressive medication	4% (9)	0% (0)	0% (0)	0.44^b^
Primary fixation of cup THA, % (n)				0.96^b^
Cemented	44% (97)	33% (7)	39% (7)	
Uncemented	36% (79)	43% (9)	39% (7)	
Cup cemented, stem uncemented	24% (43)	24% (5)	22% (4)	
Cup uncemented, stem uncemented	1% (2)	0% (0)	0% (0)	

a Kruskal-Wallis test.

b Pearson chi-square test. UPIC = unexpected positive intraoperative cultures; eGFR = estimated glomerular filtration rate.

Most cup revisions were performed because of loosening (49% [108 of 221]), instability (29% [63 of 221]), or polyethylene wear (12% [26 of 221]). A total of 96% of all cups were fixed in the acetabulum with cement. Some of the patients (7%) began empirical antibiotic treatment after cultures were obtained (Table 2). Only patients with a diagnosis of infection were subsequently treated with antibiotics for 3 months. The median follow-up was 20 months (range 0 to 54 months), with revision THA performed after a mean of 6 months (range 0 to 12 months).

**Table 2.** Results of patients who underwent cup revision for aseptic reasons at four hospitals in the NINJA network between 2015 and 2017

Variable	No UPIC (85% [n = 221])	One UPIC (8% [n = 21])	Two or more UPICs (7% [n = 18])	p value
Time between primary THA and cup revision in years, median (IQR)	12 (12.0)	11.0 (6.0)	9.0 (15.0)	0.47^a^
Indication for cup revision, % (n)				0.47^b^
Aseptic loosening of cup	49% (108)	43% (9)	56% (10)	
Instability	29% (63)	24% (5)	28% (5)	
Polyethylene wear	12% (26)	24% (5)	11% (2)	
Fracture with loosening of cup	1% (2)	5% (1)	6% (1)	
Symptomatic metal-on-metal THA	4% (9)	5% (1)	0% (0)	
Malposition of cup with impingement	6% (13)	0% (0)	0% (0)	
Intraoperative blood loss in mL, mean ± SD	602 (417)	750 (287)	738 (345)	0.27^c^
Cup revision with cemented fixation technique, % (n)	96% (211)	95% (20)	94% (17)	0.95^b^
Number of perioperative cultures harvested, mean ± SD	6 (2)	6 (2)	6 (2)	0.75^a^
Empirical antibiotics after cup revision, % (n)	6% (13)	0% (0)	22% (4)	0.01^b^
Targeted antibiotics after cup revision, % (n)	0% (0)	0% (0)	67% (12)	
Reintervention after cup revision, % (n)				
Revision of stem for any cause	3% (6)	0% (0)	17% (3)	
Revision of stem for PJI	3% (6)	0% (0)	11% (2)	
DAIR for PJI	5% (12)	5% (1)	11% (2)	0.73^b^
DAIR or revision for PJI with same microorganism	Not applicable	0% (0)	17% (3)	0.11^b^
Reposition of dislocated hip	2% (4)	0% (0)	6% (1)	0.73^b^

a Kruskal-Wallis test.

b Pearson chi-square test.

c One-way analysis of variance. UPIC = unexpected positive intraoperative cultures; DAIR = debridement, antibiotics, irrigation, and retention of implant; PJI = periprosthetic joint infection.

### Primary and Secondary Study Outcomes

The first outcome measure was the proportion of PICs in cup revision surgery. The second outcome measure was failure of the stem 2 years after cup revision, defined as revision THA for any cause or for infection. The third outcome measure was failure of the stem during follow-up according to antibiotic treatment. Patients were stratified into three cohorts: no PICs, one PIC, and two or more PICs.

The microorganisms cultured during cup revision were compared with the microorganisms cultured during subsequent additional surgeries; when these were similar, the infection was interpreted as persistent. Otherwise, it was considered a reinfection.

The baseline characteristics of the patients were noted in a case record form. Information regarding diagnostic workup, primary surgery, revision surgery, intraoperative cultures, and survival of the stem with total revision for any cause and for infection was collected.

### Bias

The four hospitals participating in this study are part of the Northern Infection Network Joint Arthroplasty in the Netherlands and use a standard diagnostic workup [14]. However, 77 patients were excluded from the analysis because an insufficient number of cultures were obtained during cup revision. An insufficient number of cultures are probably obtained in patients who were considered to have a very low prior chance of having an infection. Therefore, the percentage of PICs in our analyzed cohort may be an overestimation of the total cohort.

### Ethical Approval

The University Medical Center Groningen (UMCG) ethical board walved approval for this study. All participating hospitals are affiliated with the UMCG, and all orthopaedic surgeons are participants in the NINJA collaboration.

### Statistical Analysis

Descriptive statistics are used to describe baseline characteristics. The preoperative characteristics of the three groups were compared using the Pearson chi-square test for categorical data. For continuous data that were normally distributed, means with standard deviations were calculated and compared using the one-way analysis of variance test. In case of skewed data, median with interquartile range was used and compared using the Kruskal-Wallis test as a nonparametric ordinal approach to the one-way analysis of variance. Kaplan-Meier survival curves were created for the time from cup revision to revision THA during follow-up for all causes and for infection for all three cohorts. Additionally, Kaplan-Meier survival with revision for periprosthetic joint infection in the two or more PICs group was compared with that of a combined group of the no PIC and one PIC groups. To test the equality of survival distributions for the different cohorts, we used the log-rank (Mantel-Cox) test. Hazard ratios for revision THA in the one PIC and two or more PICs cohorts were determined using a Cox regression test and compared with those of the no PIC cohort.

SPSS version 20 was used for all analyses, and a p value < 0.05 was considered statistically significant.

## Results

### Proportion of Patients With a PIC During Cup Revision

The proportion of patients with one or more PIC was 15% (39 of 260). Of the 260 patients who underwent a cup revision, 85% had no PICs (221 of 260), 8% had one PIC (21 of 260), and 7% had two or more PICs (18 of 260). There were no differences in demographic details among these three cohorts (Table 1). In case of PIC, the microorganisms were mostly commensal skin flora such as *Staphylococcu*s species, *Cutibacterium acnes*, and *Corynebacterium* species (Table 3).

**Table 3.** Number of positive cultures in relation to the number of collected samples

Microorganism cultured	One PIC	Two or more PIC
*Corynebacterium tuberculostearicum*	0	2
*Corynebacterium afermentans*	1	1
*Corynebacterium amycolatum*	1	0
*Corynebacterium striatum*	0	1
*Cutibacterium acnes*	4	16
*Dermacoccus nishinomiyaensis*	1	0
*Enterococcus faecium*	1	0
*Escherichia coli*	0	1
*Micrococcus luteus*	2	0
*Staphylococcus aureus*	1	13
*Staphylococcus capitis*	3	8
*Staphylococcus epidermidis*	3	17
*Staphylococcus hominis*	1	0
*staphylococcus pasteuri*	0	1
*Staphylococcus saccharolyticus*	2	2
*Staphylococcus warneri*	1	0
*Streptococcus mutans*	0	2
Total positive cultures	21	64
Total number of collected samples	123	108

### Stem Survival Among Patients With No PICs, One PIC, or Two or More PICs

**Fig. 2:**
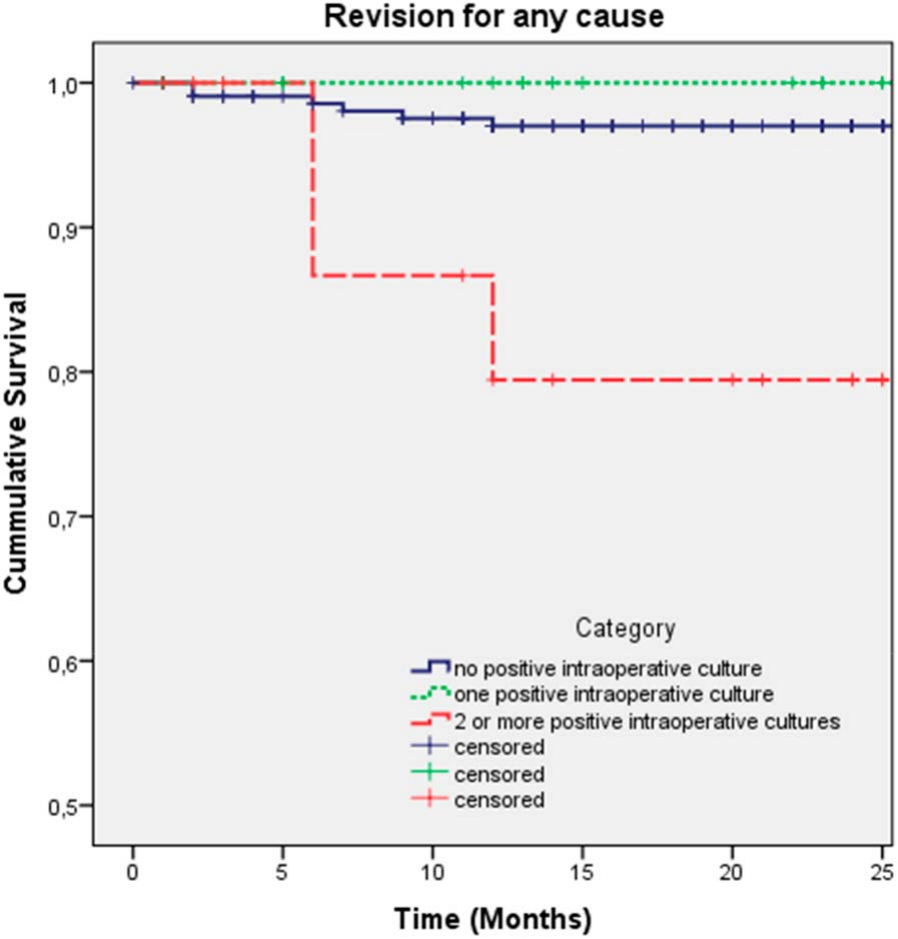
These Kaplan-Meier survival curves represent aseptic cup revision for three subgroups and revision for any cause. We performed a log-rank test of equal distribution of survival among the three cohorts (Mantel-Cox test; p = 0.22). A color image accompanies the online version of this article.

**Fig. 3:**
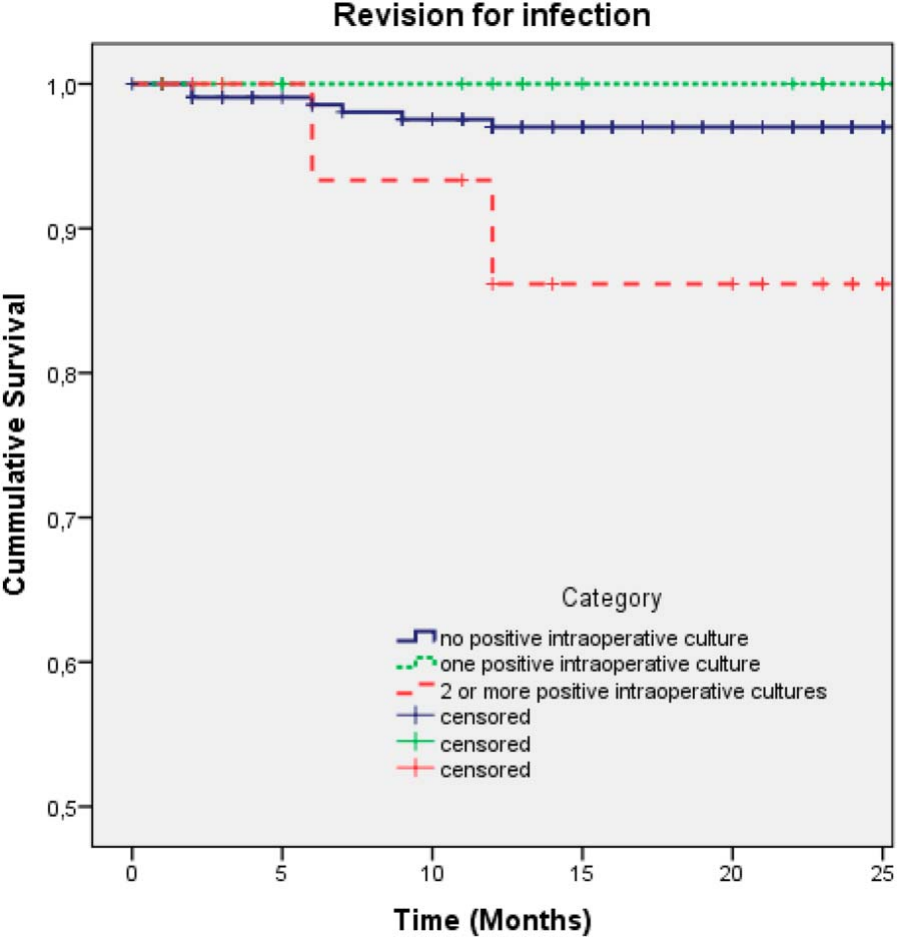
These Kaplan-Meier survival curves represent aseptic cup revision for the three subgroups and revision for periprosthetic joint infection. We performed a log-rank test of equal distribution of survival among the three cohorts (Mantel-Cox test; p = 0.18). A color image accompanies the online version of this article.

With the small numbers available, there were no differences among the study groups based on the absence or number of PICs. Regarding 2-year survival of the stem for revision for any reason, survival was 97% in the no PIC group (95% CI 95% to 99%), 100% in the one PIC group (95% CI 95% to 100%), and 79% (95% CI 59% to 100%; p < 0.01) in the two or more PICs group. There were no differences in survival of the stem with revision for periprosthetic joint infection among the study groups; 2-year survival in the no PIC group was 97% (95% CI 95% to 99%), 100% in the one PIC group, and 86% (95% CI 68% to 100%; p = 0.08) in the two or more PICs group (Table 4). The median survival of the stem was 11 years (IQR 14) and was similar among the three cohorts, irrespective of the reason for revision during the follow-up period. Hazard ratios for revision of the stem for any cause for the one PIC and two or more PICs group, compared with the no PIC cohort, was 0.23 (95% CI 0.04 to 0.58) and 0, respectively (Fig. 2). HRs for revision of the stem for an infection for the one PIC and two or more PIC groups, compared with the no PIC cohort, was 0.15 (95% CI 0.05 to 1.11) and 0, respectively (Fig. 3).

**Table 4.** Two-year survival of stem after cup revision

	Stem revision for any reason	Stem revision for PJI
No UPIC, survival (95% CI)	0.97 (0.95 to 0.99)	0.97 (0.95 to 0.99)
One UPIC, survival (95% CI)	1 (0.95 to 1)	1 (0.95 to 1)
Two or more UPIC, survival (95% CI)	0.79 (0.59 to 1)	0.86 (0.68 to 1)
Log-rank Mantel-Cox, p value	0.003	0.084

PJI = periprosthetic joint infection.

### Stem Survival According to Antibiotic Treatment of an Infected Cup

In the one PIC cohort, none of the patients were treated with antibiotics and none had revision THA with the same microorganism during the follow-up period.

In the two or more PICs cohort, 12 of 18 patients received antibiotic treatment for 3 months in the form of minocyclin (one patient), flucloxacillin (two), ciprofloxacin (two), amoxicillin (four), or clindamycin (three); additional rifampin was prescribed in five patients with staphylococci. The stem survived in one of 12 patients treated with antibiotics versus two of six patients who were not treated with antibiotics.

The patient who was treated with antibiotics underwent revision during follow-up because of persistent instability. Three periprosthetic joint infections occurred in the six patients who did not receive antibiotic treatment. One patient underwent debridement, antibiotics, irrigation, and implant retention twice to eradicate the infection. Two patients had a Girdlestone procedure during follow-up because of a periprosthetic joint infection, and one was given suppression antibiotic treatment. One of these two patients had an infection with the same microorganism as the one isolated during the primary cup revision.

## Discussion

The aim of our study was to describe the proportion of PICs in patients undergoing an isolated cup revision, determine survival of the stem during follow-up, and determine whether antibiotic treatment affected survival. Of the 260 patients in this study, the proportion of PIC was 15%; 8% (21) of patients had one PIC and 7% (18) had two or more PICs. After 2 years of follow-up, stem survival was different among the no PIC (97%), one PIC (100%), and two or more PIC groups (79%), but most of them were because of noninfectious causes. Because of the small number of patients with two or more PICs treated with antibiotics (12) and those who were not treated with antibiotics (six), no conclusion can be made about the effect of antibiotics on stem survival.

### Limitations

The most important limitation is that the number of clinically relevant PICs (two or more) was limited (18 of 260 patients; 7% of the total cohort). Although survival of the stem was similar among the three cohorts, conclusions regarding the influence or lack of influence of antibiotic treatment cannot be drawn owing to the limited sample of patients with two or more PICs. This is reflected in the large CIs. Another limitation is the follow-up period of 2 years. However, longer follow-up periods were available for 30% of patients (up to 4.5 years), but no additional failures were identified during this period (data not shown). Finally, most of the analyzed cups were cemented; thus, our results concerning the prevalence of PIC cannot be extrapolated to uncemented cups. On the other hand, because the overall risk of revision for infection is not different between cemented and uncemented THAs [2], we do not expect a difference in PIC or in survival of the stem between cemented and uncemented infected cups.

### Proportion of Patients With a PIC During Cup Revision

In our series of 260 isolated cup revisions, 15% (39) of patients demonstrated at least one PIC. To our knowledge, this is the first study reporting the presence of PIC in this patient category. Most data report on the rate of unexpected PIC in revision THAs. Recent articles reported a prevalence ranging from 10% to 33% in revision THA and TKA [3, 8, 12, 13]. A recent review reported a prevalence of 10.5% in revision THA and TKA [11]. Although we decided that the stem would not be revised, we cannot consider all PICs isolated during cup revision as unexpected, because a proportion of patients (7%) started with empirical antibiotic treatment after cultures were obtained (Table 2).

### Stem Survival Among Patients With No PICs, One PIC, or Two or More PICs

We found no difference in survival of the stem during follow-up in patients with or without PICs after 2 years of follow-up. Diagnostic criteria for periprosthetic joint infection indicate that at least two positive cultures with the same microorganism are considered a confirmed infection [7, 10]. With the numbers available, we found a difference in the risk of subsequent revision of the stem for any cause in patients who had no PIC, one PIC, or two or more PICs at the time of isolated cup revision, but not for infection. These findings are in accordance with data from revision procedures. Although studies show worse survival in patients with only one PIC than in patients with negative culture results [6, 8], other studies demonstrated that a single PIC did not result in a subsequent infection caused by the same microorganism [3, 9, 13]. Our data confirm that only one PIC is not clinically relevant; none of our patients with one PIC were treated with antibiotics, and there was no difference in survival compared with patients without PICs. Most of the patients with only one PIC had a low-virulence microorganism (such as *Cutibacteria*, coagulase-negative staphylococci, and *Corynebacteria*); thus, no conclusion can be made regarding high-virulence microorganisms such as *S. aureus* or gram-negative bacilli.

### Stem Survival According to Antibiotic Treatment of an Infected Cup

None of the patients with an infected cup were given lifelong suppressive antibiotic treatment. However, most patients in our cohort with two or more PICs were treated with antibiotics for 3 months. None of these patients experienced an infection during the follow-up period. Only six patients with two or more PICs did not receive any antibiotic treatment; three experienced an infection during the follow-up period, two of whom underwent extraction of the stem. Unfortunately, the numbers analyzed are too small to conclude about the potential protective effect of antibiotics on stem survival.

### Conclusion

When treated with antibiotics for 3 months, having more than two PICs isolated during cup revision does not have a major impact on infection survival of the stem during follow-up. Our data suggest that the stem can be left in situ when PICs occur after cup revision surgery, and lifelong suppressive antibiotic treatment is not needed for stem survival. Because the proportion of patients with PIC in our study was limited, future studies including a larger cohort of patients with a longer follow-up period are needed, in particular to conclude about potential differences between causative microorganisms.

## References

[1] Bémer P Léger J Tandé D How many samples and how many culture media to diagnose a prosthetic joint infection: a clinical and microbiological prospective multicenter study. J Clin Microbiol. 2016;2:385-391. 10.1128/JCM.02497-15 PMC4733176 26637380

[2] Hailer NP Garellick G Kärrholm J. Uncemented and cemented primary total hip arthroplasty in the Swedish Hip Arthroplasty Register. Acta Orthop. 2010;1:34-41. 10.3109/17453671003685400 PMC2856202 20180715

[3] Hipfl C Mooij W Perka C Hardt S Wassilew GI. Unexpected low-grade infections in revision hip arthroplasty for aseptic loosening: a single-institution experience of 274 hips. Bone Joint J. 2021;6:1070-1077. 10.1302/0301-620X.103B6.BJJ-2020-2002.R1 34058865

[4] Hughes RE Batra A Hallstrom BR. Arthroplasty registries around the world: valuable sources of hip implant revision risk data. Curr Rev Musculoskelet Med. 2017;2:240-252. 10.1007/s12178-017-9408-5 PMC5435639 28337731

[5] LROI: Dutch arthroplasty register. Available at: https://www.lroi.nl/. Accessed May 23, 2023.

[6] Mancheño-Losa M Lora-Tamayo J Fernández-Sampedro M Prognosis of unexpected positive intraoperative cultures in arthroplasty revision: a large multicenter cohort. J Infect. 2021;5:542-549. 10.1016/j.jinf.2021.09.001 34509512

[7] McNally M Sousa R Wouthuyzen-Bakker M The EBJIS definition of periprosthetic joint infection. Bone Joint J. 2021;1:18-25. 10.1302/0301-620X.103B1.BJJ-2020-1381.R1 PMC7954183 33380199

[8] Milandt NR Gundtoft PH Overgaard S. A single positive tissue culture increases the risk of rerevision of clinically aseptic THA: a national register study. Clin Orthop Relat Res. 2019;6:1372-1381. 10.1097/CORR.0000000000000609 PMC6554107 31136437

[9] Neufeld ME Lanting BA Shehata M Prevalence and outcomes of unexpected positive intraoperative cultures in presumed aseptic revision hip arthroplasty. J Bone Joint Surg Am. 2021;15:1392-1401. 10.2106/JBJS.20.01559 33974575

[10] Parvizi J Tan TL Goswami K The 2018 definition of periprosthetic hip and knee infection: an evidence-based and validated criteria. J Arthroplasty. 2018;5:1309-1314.e2. 10.1016/j.arth.2018.02.078 29551303

[11] Purudappa PP Sharma OP Priyavadana S Sambandam S Villafuerte JA. Unexpected positive intraoperative cultures (UPIC) in revision hip and knee arthroplasty- a review of the literature. J Orthop. 2020;17:1-6. 31879464 10.1016/j.jor.2019.06.028 PMC6919374

[12] Saleh A Guirguis A Klika AK Johnson L Higuera CA Barsoum WK. Unexpected positive intraoperative cultures in aseptic revision arthroplasty. J Arthroplasty. 2014;11:2181-2186. 10.1016/j.arth.2014.07.010 25124809

[13] Vargas-Reverón C Soriano A Fernández-Valencia JA Martínez-Pastor JC Morata L Muñoz-Mahamud E. Prevalence and impact of positive intraoperative cultures in partial hip or knee revision. J Arthroplasty. 2020;7:1912-1916. 10.1016/j.arth.2020.02.025 32147341

[14] Zijlstra WP Ploegmakers JJW Kampinga GA A protocol for periprosthetic joint infections from the northern infection network for joint arthroplasty (NINJA) in the Netherlands. Arthroplasty. 2022;1:19. 10.1186/s42836-022-00116-9 PMC8996586 35410299

